# Comparative analysis of seven types of superoxide dismutases for their ability to respond to oxidative stress in *Bombyx mori*

**DOI:** 10.1038/s41598-018-38384-8

**Published:** 2019-02-18

**Authors:** Yuta Kobayashi, Yosui Nojima, Takuma Sakamoto, Kikuo Iwabuchi, Takeru Nakazato, Hidemasa Bono, Atsushi Toyoda, Asao Fujiyama, Michael R. Kanost, Hiroko Tabunoki

**Affiliations:** 1 Department of Science of Biological Production, Graduate School of Agriculture, Tokyo University of Agriculture and Technology, 3-5-8 Saiwai-cho, Fuchu, Tokyo 183-8509 Japan; 2 Department of United Graduate School of Agricultural Science, Tokyo University of Agriculture and Technology, 3-5-8 Saiwai-cho, Fuchu, Tokyo 183-8509 Japan; 3 Database Center for Life Science (DBCLS), Joint Support-Center for Data Science Research, Research Organization of Information and Systems (ROIS), Yata 1111, Mishima, Shizuoka 411-8540 Japan; 4 Center for Information Biology, National Institute of Genetics, Yata 1111, Mishima, Shizuoka 411-8540 Japan; 5 Department of Biochemistry and Molecular Biophysics, Kansas State University, 141 Chalmers Hall, Manhattan, KS 66506-3702 USA

## Abstract

Insects are well adapted to changing environmental conditions. They have unique systems for eliminating reactive oxygen species (ROS). Superoxide dismutase (SOD) is a key enzyme that plays a primary role in removing ROS. *Bombyx mori* is a lepidopteran insect, whose body size is larger than the model insect *Drosophila melanogaster*, which enabled us to more easily examine gene expression at the tissue level. We searched *B*. *mori* SOD (BmSOD) genes using genome database, and we analyzed their function under different type of oxidative stress. Consequently, we identified four new types of BmSODs in addition to the three types already known. Two of the seven types had a unique domain architecture that has not been discovered previously in the SOD family, and they were expressed in different tissues and developmental stages. Furthermore, these BmSODs responded differently to several kinds of stressors. Our results showed that the seven types of BmSODs are likely to play different roles in *B*. *mori*; therefore, *B*. *mori* could be used to distinguish the functions of each SOD for resistance to oxidative stress that changes with the environmental conditions.

## Introduction

Lepidoptera is an order of insects that comprises 200,000 species of butterflies and moths^1^. The majority of lepidopteran insects eat plants at the larval stage and take up secondary metabolites in the process of ingesting nutrients. Some lepidopteran insects, such as the tobacco hornworm (*Manduca sexta*), striped blue crow (*Euploea mulciber*), and tiger moth (*Arctia caja*), tolerate or even utilize secondary metabolites that are toxic to other organisms, to protect against predation or for synthesis of pheromones^2–4^. However, it has been obscure how lepidopteran insects metabolized these toxic secondary metabolites including plants safely.

Reactive oxygen species (ROS) are produced in metabolic and physiological processes, such as digestion, detoxification, respiration, immune responses, and energy production^5^. Furthermore, insects are continuously exposed to diverse environmental stressors such as ultraviolet radiation, bacteria and viruses, and agrichemicals, which all can result in production of ROS^6^. Thus, insects are continually subjected to oxidative stress from ROS.

ROS are free radicals, such as superoxide anion, hydroxyl, nitric oxide radical, hydrogen peroxide, and singlet oxygen^5^. At the cellular level, excessive ROS can damage DNA, proteins, and lipids. We are interested in the molecular evolutionary adaptations that lepidopteran insects might have to control levels of ROS, conferring ability to adjust to and survive environmental changes, including the mechanisms that lepidopteran insects employ to resist various oxidative stressors.

The genome sequences of several lepidopteran insects have been annotated by combinations of automated and manual methods., and researchers can now access public databases containing the genomic information of lepidopteran insects for comparative analyses^7^. However, much work remains, to experimentally characterize the functions of gene products identified in genome databases. Superoxide dismutase (SOD) has been identified in lepidopteran model insects, as reported in the EnsemblMetazoa database (https://metazoa.ensembl.org/). SOD is an important enzyme with a primary role of eliminating superoxide anions^8^. Three types of SOD proteins have previously been reported in insects: SOD1 is a major cytoplasmic antioxidant enzyme that metabolizes superoxide radicals to molecular oxygen and hydrogen peroxide, thus providing a defense against oxygen toxicity. SOD2 is a mitochondrial matrix enzyme that scavenges oxygen radicals produced by the extensive oxidation–reduction and electron transport reactions that occur in the mitochondria. These SOD proteins are classified as copper/zinc SOD (Cu/Zn SOD; SOD1) and manganese SOD (Mn SOD; SOD2), respectively. In addition, an extracellular (EC) form of SOD protein has been identified. EC-SOD (SOD3) is found mainly in the hemolymph and molting fluid of insects^9^. Although additional SODs with a unique structure have been discovered, including a membrane-bound form in *Caenorhabditis elegans*, their functions have not been clarified^10^.

The silkworm. *Bombyx mori*, is an intensively studied lepidopteran insect that eats mulberry leaves. It is much larger than *Drosophila melanogaster*, so by using this species, we can more easily examine the expression of protein or mRNA in individual tissues. We are interested in the molecular mechanisms by which this species adapts to the environment, including the physiological functions of its SODs.

In a previous study, we reported that SOD1 and SOD2 proteins in *B*. *mori* play crucial roles in eliminating ROS throughout this species’ life cycle^11^. In addition, *B*. *mori* SOD1 (BmSOD1) and SOD2 (BmSOD2) proteins showed different expression patterns in each tissue of fifth-instar larvae. Thus, these BmSODs might have tissue-specific roles during development.

In this study, we identified and characterized four new types of SODs in *B*. *mori* and compared their mRNA expression by RNA-seq and analyzed their expression under several kinds of oxidative stress, in comparison with those of previously known types of SODs in this species.

## Results

### Seven types of superoxide dismutase were identified from *B*. *mori* datasets

First, we searched for SODs in the *B*. *mori* genome, using their SOD motifs from the Ensembl dataset. We found the three known *B*. *mori* SOD genes and four types of new SOD amino acid sequences. To confirm the expression of these new sequences in *B*. *mori*, we performed cDNA cloning of the four new types of *B*. *mori* SODs from larval tissues. We named the four types of SODs as BmSOD4, BmSOD5, BmSOD6, and *B*. *mori* copper chaperone (BmCCS) because of its similarity to *C*. *elegans* SOD or *H*. *sapience* CCS. (WormBase; https://www.wormbase.org/)^12^. The deduced open reading frame (ORF) of BmSOD4 was 600 nucleotides, encoding a protein of 201 amino acids, molecular weight of 21,173 Da, and a putative isoelectric point (pI) of 5.87. The deduced ORF of BmSOD5 was 729 nucleotides, encoding a protein of 242 amino acids, molecular weight of 26,107 Da, and pI of 5.70. The deduced ORF of BmSOD6 was 3,399 nucleotides, encoding a protein with 1,471 amino acids, molecular weight of 166,849 Da, and pI of 6.16. The deduced ORF of BmCCS was 675 nucleotides, encoding a protein of 224 amino acids, molecular weight of 22,719 Da, and pI of 6.94.

**Figure 1:**
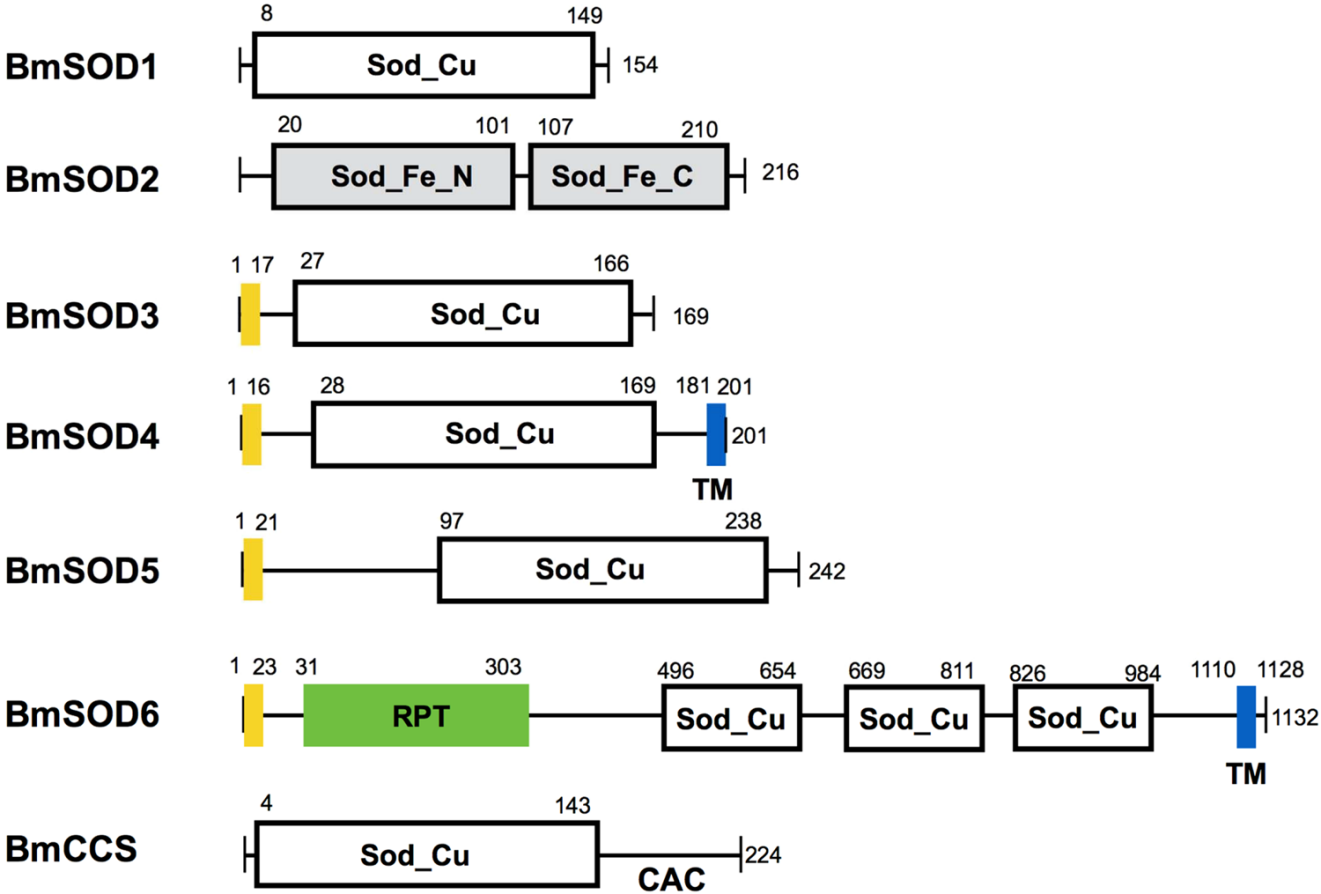
Putative domain structure of *Bombyx mori* superoxide dismutases (BmSOD)-1 to BmSOD 6 and Bm copper chaperone for superoxide dismutase I(-CCS). Upper digits show the amino acid position. Sod_cu and Sod_Fe are the distinguishing SOD domains. Yellow boxes, signal peptide; blue boxes, transmembrane domain; and green box, repeat (RPT) domain.

A protein motif search revealed that BmSOD4 contains a 16-residue secretion signal sequence, a copper/zinc superoxide dismutase domain (Sod_Cu, pfam; PF00080) at position A28-I169, and a transmembrane segment at V181-N201. BmSOD5 contains a 21-residue secretion signal sequence, and a Sod_Cu domain at position A97-I238. BmSOD6 contains a 23-residue secretion signal sequence, a repeat (RPT) domain at L31-L303, three Sod_Cu domains at positions Y496-V654, G669-Y811, and H826-I984, and transmembrane segment at P1110-E1128. BmCCS contains a Sod_Cu domain at position D4-I143 and at the C-terminal a carnitine/acylcarnitine carrier (CAC) motif (Fig. 1). The sequences of these BmSOD cDNAs cloned from fat body mRNA were consistent with the predicted sequences that we identified from the genome database.

In addition, we checked corresponding genomic regions using the genome browser in SilkBase. We identified the location of the BmSOD1 gene to four exons between 784,426 and 787,928 bp on chromosome 23, that of BmSOD2 gene to five exons between 3,819,813 and 3,824,147 bp on chromosome 3, of BmSOD3 gene to three exons between 15,258,942 and 15,270,235 bp on chromosome 10, of BmSOD4 gene to one exons between 10,520,085 and 10,520,690 bp on chromosome 8, of BmSOD5 gene to six exons between 15,288,397 and 15,296,246 bp on chromosome 10, of BmSOD6 gene to 23 exons between 7,235,573 and 7,252,862 bp on chromosome 26, and of BmCCS gene to four exons between 431,024 and 433,982 bp on chromosome 4. The nucleotide sequences of four new BmSOD cDNAs reported here have been submitted to GenBank/DDBJ Accession Nos. LC229590, LC229591, LC229592, and LC229593.

**Figure 2:**
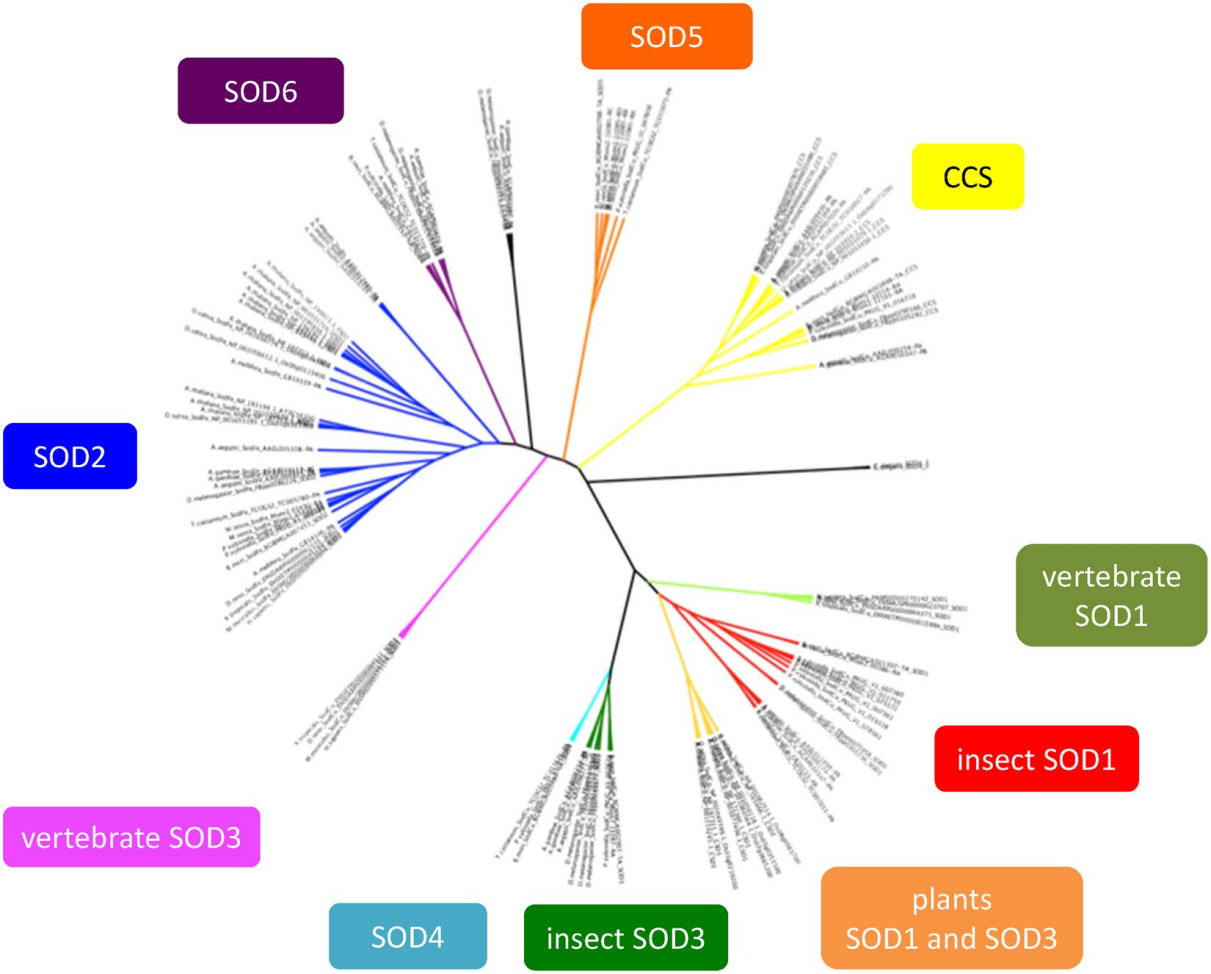
Phylogenetic tree of seven types of *Bombyx mori* superoxide dismutases (SODs) and the SOD proteins of other species. The amino acid sequences of the SODs used in this study are showed in Supplementary Table 1.

In a phylogenetic tree that contains amino acid sequences of BmSODs and SODs of some other species shown in Supplementary Table 1, the seven identified BmSODs and SODs of other species were distributed among 10 clusters (Fig. 2). BmSOD1 was located in the insect SOD1 cluster. Interestingly, BmSOD4–BmSOD6 were distributed in a cluster that contained mainly insect species (Fig. 2). BmSOD4 had > 89% amino acid sequence identity to *Plutella xyllostella* (PXUG_V1_011049), BmSOD5 was most similar to *M*. *sexta* SOD5 (Msex2_11085-RC: 99%, Msex2_11086-RD: 95%, Msex2_11087-RA: 82%, Msex2_11088-RB: 98%), and *P*. *xyllostella* (PXUG_V1_047659: 96%). BmSOD6 was most similar to genes from *Anopheles gambiae* (AGAP001623-PA: 84%, AGAP007497-PA: 57%), *D*. *melanogaster* (FBpp0288490: 86%, FBpp0288489: 86%), *Pullucella xylostella* (PXUG_007562: 96%), and *Tribolium castaneum* (TC011770-PA: 87%). Transmembrane domain found only in BmSOD4 and BmSOD6 amino acid sequences.

### Developmental stage and tissue-specific expression patterns of BmSOD mRNAs

**Figure 3:**
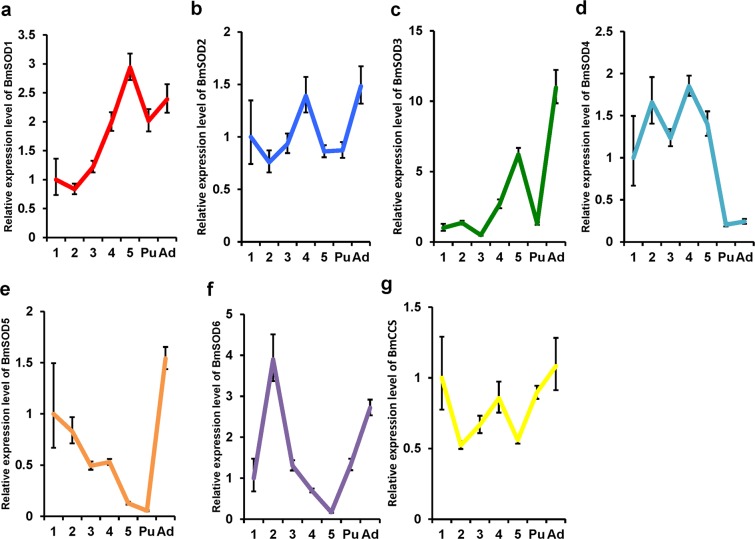
Developmental stage distribution of *Bombyx mori* superoxide dismutases (BmSODs). 1; Day zero of the first instar larvae (n = 3), 2; second instar larvae (n = 3), 3; third instar larvae (n = 3), 4; fourth instar larvae (n = 3), and 5; fifth instar larvae (n = 3), and Pu of the pupae (n = 3) and Ad; adult (n = 3). mRNA expression in the entire body of these samples are relative quantification (RQ) values. RQ values represent the relative expression levels calculated using day zero of first instar larvae sample as 1. Error bars represent the relative minimum/maximum expression levels of the mean RQ value. Bmrp49 was use as the endogenous control. Technical replication was performed in triplicate.

We investigated the distribution of BmSOD mRNAs during the developmental stages by quantitative RT-PCR (Fig. 3.) The expression of BmSOD1 mRNA gradually increased in whole larval bodies during larval development (Fig. 3a). The expression of BmSOD2 mRNA slightly fluctuated throughout the developmental stages (Fig. 3b). The expression of BmSOD3 mRNA increased dramatically in the fifth larva instar l and adult whole bodies compared with the first-instar larval whole bodies (Fig. 3c). The expression of BmSOD4 mRNA decreased in the pupa and adult whole bodies compared with the first-instar larval whole bodies (Fig. 3d). The expression of BmSOD5 mRNA gradually decreased from the first instar larva to pupa; however, the expression of BmSOD5 mRNA increased in the adult (Fig. 3e). The expression of BmSOD6 mRNA increased in the second instar of the whole larval bodies, but decreased in the fifth instar larvae, and BmSOD6 mRNA increased in the pupae and adult whole bodies (Fig. 3f). The expression of BmCCS mRNA decreased in the second and fifth instar of the larval whole bodies but recovered in the adult developmental stages (Fig. 3g).

**Figure 4:**
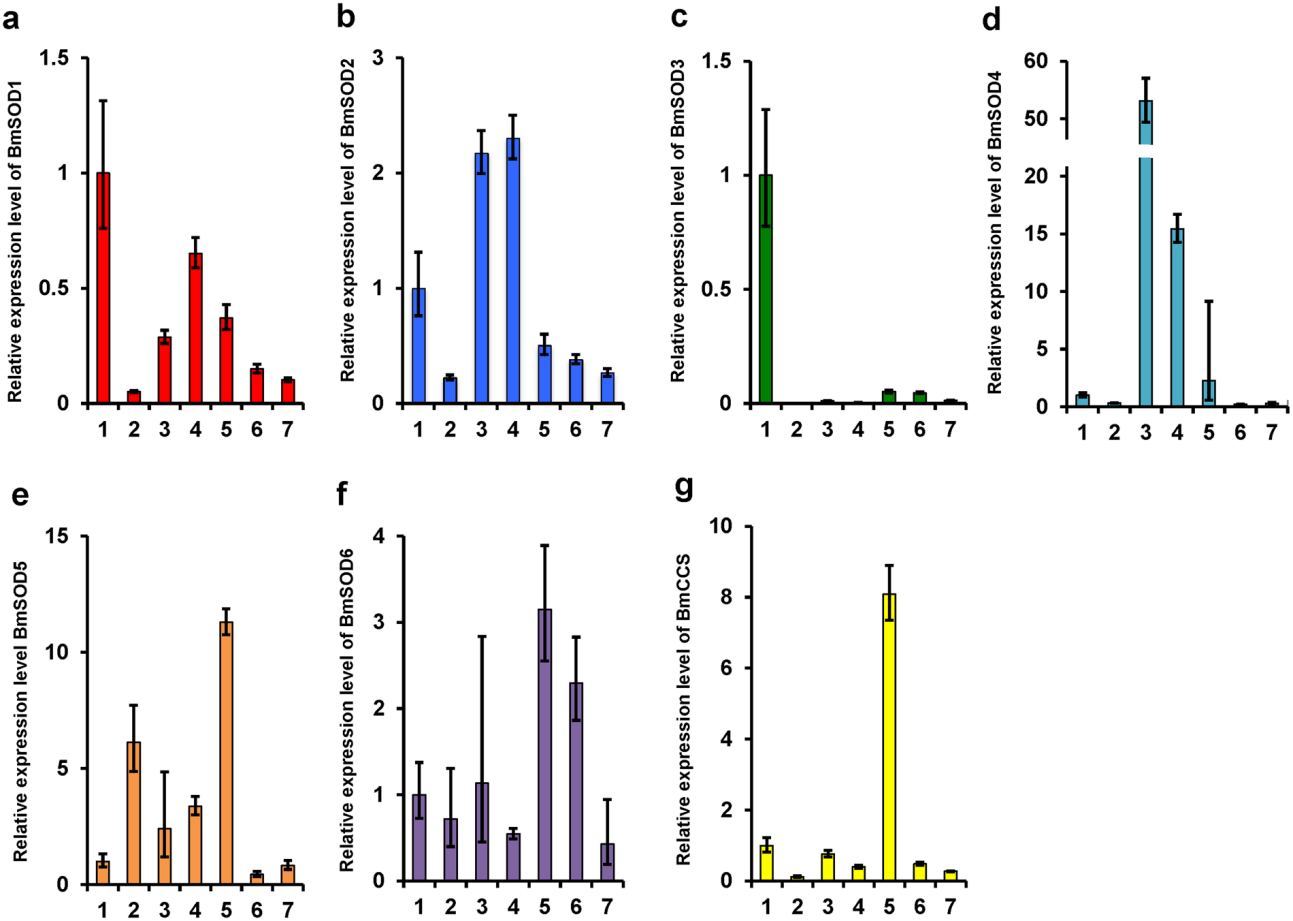
Tissue distribution of *Bombyx mori* superoxide dismutases (BmSODs). 1; Fat body (n = 3), 2; silk gland (n = 3), 3; midgut (n = 3), 4; Malpighian tubules (n = 3), 5; testis (n = 3), 6; ovary (n = 3), and 7; hemocytes (n = 3). Relative mRNA expressions in each tissue were relative quantification (RQ) values. RQ values represent the relative expression levels calculated using the fat body sample as 1. Error bars represent the relative minimum/maximum expression levels of the mean RQ values. Bmrp49 was used as the endogenous control. Technical replication was performed in triplicate.

We further investigated the distribution of the BmSOD mRNAs in different tissues and compared them to BmSOD mRNA expression in the fat body (Fig. 4). All BmSOD mRNAs were expressed in all tissue samples. BmSOD1 mRNA was expressed mainly in the fat body and Malpighian tubules (Fig. 4a); BmSOD2 mRNA was expressed mainly in the midgut and Malpighian tubules (Fig. 4b); BmSOD3 mRNA was expressed mainly in the fat body (Fig. 4c); BmSOD4 mRNA was expressed mainly in the midgut, Malpighian tubules, and testes (Fig. 4d); BmSOD5 mRNA was expressed mainly in the testes, silk glands, Malpighian tubules, and midgut (Fig. 4e); BmSOD6 mRNA was expressed mainly in the testes and ovaries (Fig. 4f); and BmCCS mRNA was expressed mainly in the testes (Fig. 4g). All BmSODs differed in their tissue-specific and developmental expression patterns.

### Analysis of the expression of BmSOD mRNAs by RNA-seq

To examine in more detail the expression of each BmSOD at the individual level, we used RNAseq to investigated the expression of BmSOD mRNAs in five tissues in *B*. *mori*, calculating fragments per kilobase of transcript per million (FPKM) mapped reads (Table 1). BmSOD1 and BmSOD2 mRNAs had comparatively high levels of expression in each tissue, whereas BmSOD3, BmSOD4, and BmCCS mRNAs had tissue-specific expression patterns. Both BmSOD5 and BmSOD6 displayed relatively low expression values in these tissues.

**Table 1:** FPKM value of *B*. *mori* SODs in the five tissues.

BmSODs	Fat body	Midgut	Malpighian tuble	Silk glands	Testis
BmSOD1	1322.4	160.2	3425.7	1586.3	655.2
BmSOD2	91.8	94.5	1182.6	877.9	202.1
BmSOD3	1.4	72.4	1.4	0	5.2
BmSOD4	0.1	125.7	230.6	0.3	0.3
BmSOD5	0	0.2	0	0.3	26.9
BmSOD6	0	0.2	0.7	0	1.1
BmCCS	13.6	12.4	36.0	4.9	560.2

### Induction of oxidative stress by ultraviolet radiation

**Figure 5:**
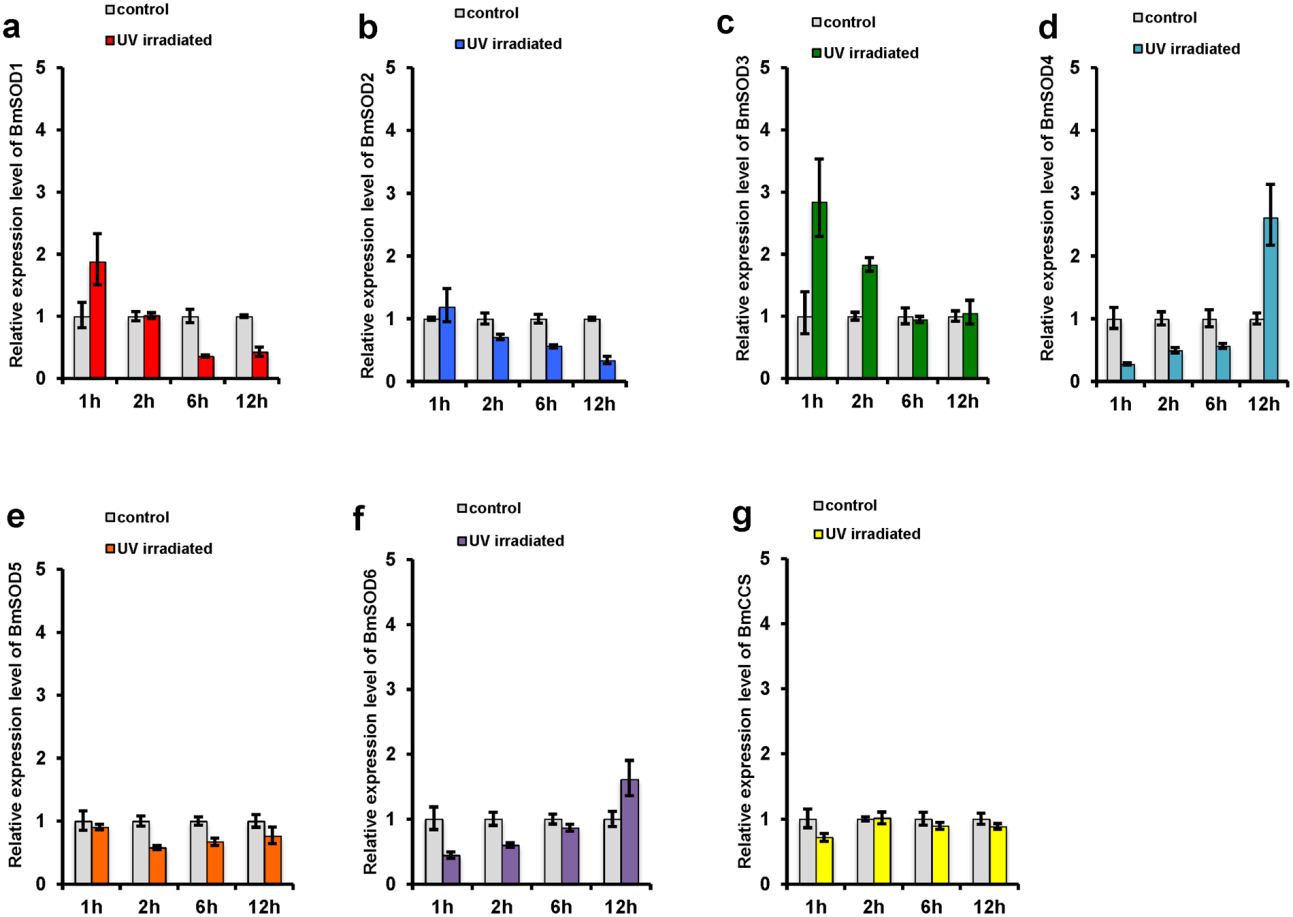
*Bombyx mori* superoxide dismutases (BmSODs) mRNA expression in ultraviolet (UV)-irradiated fat body. (**a**) BmSOD1, (**b**) BmSOD2, (**c**) BmSOD3, (**d**) BmSOD4, (**e**) BmSOD5, (**f**) BmSOD6, and (**g**) BmCCS mRNA expressions in the fat bodies pooled from larvae subjected to UV irradiation at 1.0 h (n = 3), 2.0 h (n = 3), 6.0 h (n = 3), and 12.0 h (n = 3) and of non-irradiated controls (n = 3 each) were plotted as relative quantification (RQ) values. Error bars indicate the relative minimum/maximum expression levels against mean RQ values. Bmrp49 was used as the endogenous control. Technical replication was performed in triplicate.

To examine the expression of the seven types of BmSODs under oxidative stress in *B*. *mori*, we induced three types of oxidative stress in the larvae under experimental conditions. First, we examined the response of BmSOD mRNAs in the fat body using quantitative reverse transcription polymerase chain reaction (qRT-PCR) after ultraviolet (UV) irradiation for 1, 2, 6, and 12 h. In the fat body, BmSOD1 mRNA expression increased at 1 h and decreased at 6 h and 12 h (Fig. 5a). BmSOD2 mRNA expression decreased in a UV-exposed time-dependent manner (Fig. 5b). BmSOD3 mRNA expression increased at 1 h and 2 h (Fig. 5c). BmSOD4 mRNA expression increased in a UV-exposed time-dependent manner (Fig. 5d). BmSOD5, BmSOD6, and BmCCS mRNA expressions were not significantly changed after UV-irradiation (Fig. 5e–g).

### Induction of oxidative stress by rotenone

**Figure 6:**
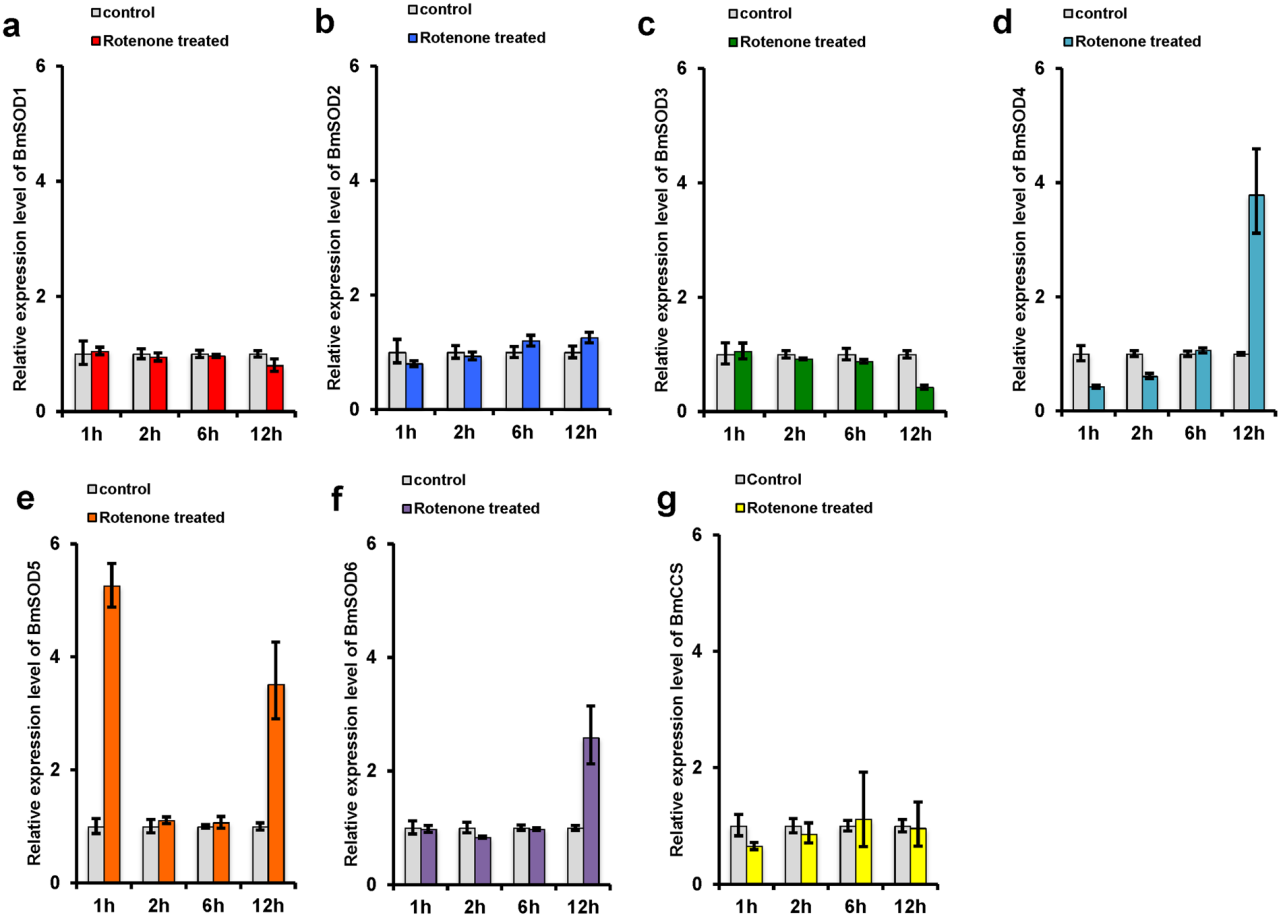
*Bombyx mori* superoxide dismutases (BmSODs) mRNA expression in rotenone (ROT)-injected fat body. (**a**) BmSOD1, (**b**) BmSOD2, (**c**) BmSOD3, (**d**) BmSOD4, (**e**) BmSOD5, (**f**) BmSOD6, and (**g**) BmCCS mRNA expressions in fat bodies pooled from larvae subjected to ROT treatment at 1.0 h (n = 3), 2.0 h (n = 3), 6.0 h (n = 3), and 12.0 h (n = 3) and in controls (n = 3 each) were plotted as relative quantification (RQ) values. Error bars indicate the relative minimum/maximum expression levels against the mean RQ values. Bmrp49 was used as the endogenous control. Technical replication was performed in triplicate.

Rotenone was used to produce a chemical oxidative stress, to examine the effect of BmSOD mRNA expression. Rotenone is a mitochondrion complex I inhibitor, which accelerates ROS production in the cell^13^. Based on computer simulations of reactivity using JMP (https://www.jmp.com), we determined 25.0% of the lethal dose (LD) of rotenone for the day-three fifth instar larvae to be 4.52 µg/g (LD_25_; 95.0% confidence interval [CI], 1.46–7.61) and examined the expression of the BmSOD mRNAs using qRT-PCR at 1, 2, 6, and 12 h after injecting rotenone at this concentration into day 3 fifth instar larvae. BmSOD1-3 and BmCCS mRNA expression was not altered at any time after rotenone injection (Fig. 6a–c,g); however, BmSOD4 and BmSOD6 mRNA expression significantly increased at 12 h after injection of rotenone (Fig. 6d,f). BmSOD5 mRNA expression increased at 1 h and 12 h after injection of rotenone (Fig. 6e).

### Induction of oxidative stress by injection of *Micrococcus luteus*

**Figure 7:**
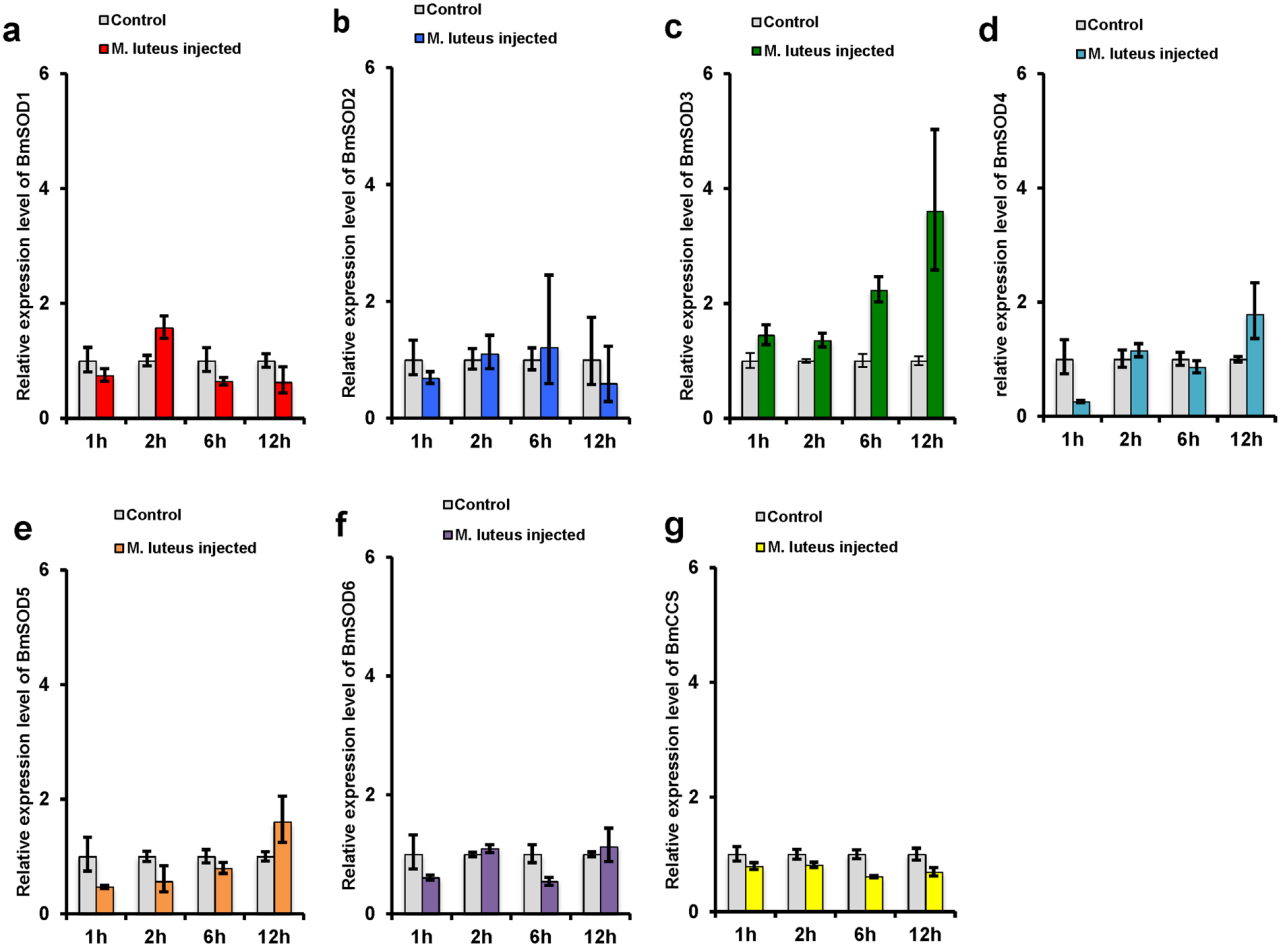
*Bombyx mori* superoxide dismutases (BmSODs) mRNA expressions in *Micrococcus luteus-*injected fat bodies. (**a**) BmSOD1, (**b**) BmSOD2, (**c**) BmSOD3, (**d**) BmSOD4, (**e**) BmSOD5, (**f**) BmSOD6, and (**g**) BmCCS mRNA expressions in fat bodies pooled from larvae subjected to *M*. *luteus* injected at 1.0 h (n = 3), 2.0 h (n = 3), 6.0 h (n = 3), and 12.0 h (n = 3) and in controls (n = 3 each) were plotted as relative quantification (RQ) values. Error bars indicate the relative minimum/maximum expression levels against the mean RQ values. Bmrp49 was used as the endogenous control. Technical replication was performed triplicate.

Oxidative stress occurs during the immune response to bacteria in insects, because ROS are a secondary product by melanization promoted by phenoloxidase in response to infection. Fat body plays a crucial role in producing proteins for the insect’s immune system; therefore, we examined the expression pattern of BmSOD mRNAs using qRT-PCR in fat body at 1 h, 2 h, 6 h, and 12 h after injecting *M*. *luteus*. BmSOD1,-2,-4,-5, and-6 and BmCCS mRNA expression did not significantly change at any time after injection of *M*. *luteus* (Fig. 7a,b,d,e–g). However, BmSOD3 mRNA level increased at 6 h and 12 h after injection of the bacteria (Fig. 7c).

## Discussion

### BmSODs were categorized into seven types

In this study, we characterized previously unknown types of BmSODs by comparing protein domain architecture and their expression patterns. We found four new gene sequences from the *B*. *mori* genome database that belong to the SOD family and for which the functions in insects have been obscure.

cDNA cloning, phylogenic tree analysis, and protein motif analysis showed that *B*. *mori* SODs can be categorized as seven types, four of which were new. BmSOD3 and BmSOD5 genes are near each other on the same chromosome, however, these genes differ in encoded protein structure and expression pattern. BmSOD3 has previously been known as TIME-EA4, the function of which was annotated for controlling diapause, which helps to delay development during severe environmental conditions^14,15^.

BmSOD4 has a secretion signal at the N-terminal and a transmembrane segment at the C- terminal region. One previously identified membrane-bound SOD that has a C-terminal transmembrane segment was discovered as a splicing variant of a SOD in *Caenorhabditis*. *elegans*^10^. *Caenorhabditis elegans* membrane-bound SOD was present in the plasma membrane; however, its function is not clear. In addition, *Drosophila* SOD3 (DmSOD3) occurs as four variants. DmSOD3 variant 2 has a hydrophobic terminal sequence at the C-terminal^16^ and the phylogenic tree showed that BmSOD4 occurs in a cluster near invertebrate SOD3; therefore, BmSOD4 might have evolved after duplication of BmSOD3, with addition of a C-terminal transmembrane region.

We found that BmSOD6 had three Sod_Cu domains and had the longest transcript of all BmSODs. Furthermore, we found BmSOD6 types of SODs in other insect genome datasets, such as *Anopheles gambiae*, *D*. *melanogaster*, *T*. *castaneum*, *Plutella xylostella*, and *Apis mellifera;* however, only the BmSOD6 sequence was predicted to contain the transmembrane domain at the C-terminal region in these insect species.

Human CCS has been reported as a major player in SOD1 copper loading^17^. Thus, BmCCS might play a major role in BmSOD1 copper loading.

### Stage and tissue distribution of BmSODs

BmSOD mRNAs showed unique developmental stage-specific expression patterns. Expression levels of all BmSOD mRNAs commonly decreased from the late-larval to pupal-development stages (Fig. 3) During the late-larval to prepupal developmental stages in *B*. *mori*, autophagy and apoptosis occur mainly in the midgut, especially during the early pupal developmental period, for dynamic remodeling of the body structure into the adult^18^. As the concentration of ecdysteroids increases in the hemolymph at the late-larval stage, pupation is initiated^19^. The neuropeptide prothoracicotropic hormone (PTTH) increases from specific physiological stimuli to produce and release ecdysteroids from the prothoractic gland. ROS is then produced through the ecdysteroid signaling pathway, which is stimulated by secretion of PTTH^20^. Our results showed that the expression levels of all BmSODs decreased from the late-larval to the pupal development stages, which coincides with the increased concentration of ecdysteroids in the haemolymph^19^. It has also been reported that ROS production is increased through the ecdysteroid signaling pathway in *B*. *mori*^20^. Our previous study showed that the BmSOD2 protein expression levels were significantly decreased in the fat body before pupation and that ecdysone is related to control of the expression of BmSOD2 proteins^11^. Even though BmSODs play a role in removing ROS, their expression may decrease during pupation to enable a physiologically useful increase in ROS level. Therefore, the expression of BmSODs might be controlled in relation to the apoptosis stimulated by ROS when *B*. *mori* larval tissue is restructured during metamorphosis. More study is needed to clearly define the relationship between BmSODs and morphological changes occurring during pupation.

BmSOD4 was mainly expressed in the midgut and Malpighian tubules rather than in the fat body during the larval period. *B*. *mori* feed only on mulberry leaves, which contain three types of sugar-mimic alkaloids-1,4-dideoxy-1,4-imino-D-arabinitol, 1-deoxynojirimycin, and 1,4-dideoxy-1,4-imino-D-ribitol^21^. These sugar-mimicking alkaloids inhibit larval growth in other mulberry specialists; however, *B*. *mori* larvae might have enzymes that are responsible for metabolising these alkaloids^22^. It was speculated that this metabolic process could contribute to ROS generation, because ATP may be necessary to metabolize these alkaloids in the midgut and Malpighian tubules^23^. Therefore, BmSOD4 would play a role in removing generated ROS when sugar-mimicking alkaloids contained in mulberry leaves are metabolized.

BmSOD5, BmSOD6, and BmCCS mRNAs were expressed mainly in the testes of the day 3 fifth instar larvae. *B*. *mori* has two types of sperm; apyrene and eupyrene^24,25^. Both sperm types are essential for fertilization in *B*. *mori*; however, the role of this evolutionarily conserved system in fertilization remains unclear. The differentiation of spermatogonia is initiated during the second instar larva stage of *B*. *mori* and apyrene sperm development is activated from the late larval–development stage. The differentiation of spermatogonia also needs ATP for energy^26^. This process also increases ROS production. Thus, these BmSODs might play a role in removing ROS generated during spermatogenesis.

### The response of seven types of BmSODs to different types of stressors

Next, we investigated the expression of seven types of BmSODs in response to different type of stressors. First, *B*. *mori* larvae were exposed to UV irradiation. Exposure to UV radiation induces oxygen radical generation through damage to the biological molecules^27^. In this experiment, each BmSOD mRNA showed a different expression pattern in the time course following exposure or among fat body, midgut, integument, Malpighian tubules (Fig. 5 and Supplementary Figs 1–3). Expression of BmSOD2 mRNA did not increase under any UV-irradiation conditions in the current study; however, its expression increased after UV irradiation of 16 h in our previous study. In our previous experiment^11^, we kept UV-irradiated *B*. *mori* larvae for 16 h in the insectary until the fat body samples were dissected, and it might be that there was induction of BmSOD2 expression from photoreactivation.

We then investigated BmSOD mRNA expression after injection of rotenone into larvae. Rotenone induces the production of ROS by inhibiting mitochondrion complex I^13^. BmSOD4, BmSOD5 and BmSOD6 mRNAs in the fat body temporarily responded to rotenone injection. BmSOD4 in the midgut did not respond to rotenone injection (Supplementary Fig. 4). BmSOD1 and BmSOD2 mRNAs in both fat body and midgut did not respond to rotenone injection. In the previous study, BmSOD1 and BmSOD2 proteins in the BmN4 cell line did not respond to rotenone stimuli^11^, and these data correspond to those from our previous study.

Finally, we investigated BmSOD mRNA expression after the *M*. *luteus* injection. *M*. *luteus* induces melanization as an immune response, with the production of ROS in the insect hemolymph^28^. ROS are produced as a secondary metabolite during melanization, helping to kill the bacteria^28^. In this experiment, only BmSOD3 mRNA increased in level after the *M*. *luteus* injection, and its expression increased in a time-dependent manner. Thus, BmSOD3 may help to protect host tissue from damage caused by SODs produced during the immune response. Conversely, a study showed that low SOD3 activity in mice accelerated pathogen clearance from the liver by the neutrophils, and SOD3 might alter an innate immune response by suppressing the production of TNF-α^29^. Our results are consistent with a system in *B*.*mori* poised to deal with ROS generated produced by different stressors, with each BmSOD playing a role for removing superoxide generated in different tissues.

### Comparison of SODs between *B*. *mori* and *M*. *sexta*

To compare the characteristics of SODs between *B*. *mori* and *M*. *sexta*, both lepidopteran insects that are models for biochemical research, we also searched the *M*. *sexta* genome database^30^ for SOD genes. The *M*. *sexta* larvae, known as tobacco hornworms, feed on plants in the family *Solanaceae*, which produce toxic allelochemicals including nicotine and other neurotoxic alkaloids^2^. *M*. *sexta* has five types of SODs (Supplementary Table 2 and Supplementary Fig. 5). The expression trends were similar to those of BmSODs from FPKM data (Table 1, Supplementary Table 3); therefore, BmSOD and MsSOD can be categorized into major- and minor-expressed SODs. Minor-expressed SODs might be considered to have an auxiliary role in the major-expressed SODs, such as SOD1, SOD2, and SOD3. And, *M*. *sexta* did not have BmSOD4 or BmSOD6 type SODs.

Interestingly, the FPKM value of MsSOD3 also increased in the fat body after injection of larvae with *M*. *luteus* (Supplementary Fig. 6). The mRNA expression levels of BmSOD3 and MsSOD3 might need to be increased to protect the host from ROS generated during the killing of bacteria by the innate immune system; therefore, the function of SOD3 would be evolutionally conserved in the innate immune systems in *B*. *mori* and *M*. *sexta*.

BmSOD3 and MsSOD3 are predicted to be proteins secreted into the hemolymph because they have a secretory signal in their amino acid sequence. Thus, BmSOD3 and MsSOD3 respond to the stimulus of *M*. *luteus*, and it is predicted that BmSOD3 and MsSOD3 are rapidly secreted from fat body into the hemolymph in order to remove ROS generated by the immune response. Although BmSOD5 is also predicted to be a secretory protein, BmSOD5 differed from BmSOD3 in terms of the response to stressors that it exhibited.

## Conclusion

BmSODs play a role in controlling the amount of ROS in different tissues of the body or during different developmental stages. Through a series of experiments, we found that the responsive ability of the seven types of BmSODs to different types of oxidative stress differed. By searching the genome database, we found unique and previously undescribed types of SODs in this study. Thus, *B*. *mori* might have a system to deal with the ROS generated by different physiological and external stressors, with BmSOD playing a role in removing ROS generated in different tissues. However, it has remained unclear why insects have many SODs compared with vertebrate species. In future study, we will measure the ROS levels in particular tissues and evaluate the relationship of these SODs at the protein level with ROS, in order to elucidate the molecular mechanisms controlling ROS in *B*. *mori*.

## Materials and Methods

### Insects

The *B*. *mori* eggs were supplied by Ueda-Sha Co. Ltd. (Nagano, Japan). Silkworm larvae were reared on the artificial diet silk-mate 2S (Nosan, Tsukuba, Japan). Insects were maintained at 25 °C with a 12-h light/dark cycle.

The *B*. *mori* strain o751 (wild-type) used in the RNA-seq analysis was obtained from the Institute of Genetic Resources, Faculty of Agriculture, Kyushu University (NBRP silkworm database: http://silkworm.nbrp.jp/index_en.html).

### Identification of *B*. *mori* superoxide dismutase sequences by HMM search

The HMM search program in the HMMER package (version 3.1b1)^31^ was used to detect SOD candidates. HMM profiles of the Copper/zinc superoxide dismutase domain (Sod_Cu, PF00080), Iron/manganese superoxide dismutases, alpha-hairpin domain (Sod_Fe_N, PF00081) and Iron/manganese superoxide dismutases, C-terminal domain (Sod_Fe_C, PF02777) in the Pfam 27.0 database^32^ were queried against deduced protein sequences in a *B*. *mori* Ensembl Gene dataset^33^ with default parameters. Also, we made BLAST search against the assembled genome (2017) in SilkBase (http://silkbase.ab.a.u-tokyo.ac.jp/cgi-bin/index.cgi) and then checked corresponding genome positions utilizing genome browser in SilkBase.

SOD orthologs of the following species were obtained from public databases: *O*. *sativa*, *A*. *thaliana* (Protein: http://www.ncbi.nlm.nih.gov/protein), *H*. *sapiens*, *M*. *musclus*, *Xenopus*, *Danio rerio* Ensemble (http://www.ensembl.org/index.html), *D*. *melanogaster*, *A*. *gambiae*, *A*. *aegypti*, *A*. *mellifera*, *T*. *castaneum* (Ensembl Metazoa: http://metazoa.ensembl.org/index.html), *P*. *xylostella* (KONAGAbase: http://dbm.dna.affrc.go.jp/px/), *M*. *sexta* (ManducaBase: http://agripestbase.org/manduca/). A phylogenetic analysis was performed using Clustal W ver. 2.1 web interface at DDBJ (http://clustalw.ddbj.nig.ac.jp/). A protein motif search was conducted using SMART (http://smart.embl-heidelberg.de/). The alignment of the BmSOD amino acid sequences and SOD orthologs from other species was conducted using CLC Sequence viewer 7.0 (CLC Bio Japan Inc. Tokyo, Japan). All analyses were performed with default parameters for the software.

### Purification of total RNA and cDNA synthesis from different tissues and whole-body samples

Various tissues were dissected from day 3 fifth-instar larvae: midgut, silk gland, fat body, Malpighian tubules, hemocytes, testis, and ovary (n = 3 each). These tissues were stored at −80 °C until use. 1–5 instar larval, pupal, and adult whole bodies were also used for total RNA purification. Whole bodies (n = 3 each) were crushed with liquid nitrogen using a mortar and pestle. Tissues and whole bodies were weighed and homogenized with lysis buffer from a PureLink® RNA extraction kit (Thermo Fisher Scientific Inc., Valencia, CA, USA) and then centrifuged at 13,000 × g for 10 min. Next, the supernatants were collected and processed for RNA purification according to the manufacturer’s instructions. Purified total RNA (1 μg) was processed for qRT-PCR or cDNA synthesis using a PrimeScriptTM 1st strand cDNA Synthesis Kit (Takara Co. Ltd., Tokyo, Japan).

### cDNA cloning of *B*. *mori* SODs

cDNA was synthesized using total RNA (1 μg) from the fat body of fifth instar larvae. Primers designed for cDNA cloning of novel BmSODs nucleotide sequence are shown Supplementary Table 4. These cDNA sequences were amplified by PCR using KOD–plus-neo polymerase (Toyobo Co. Ltd., Tokyo, Japan) with specific primers (Supplementary Table 4). The amplified products were cloned into the cloning vector PCR 2.1 Topo (Invitrogen, Van Allen Way, Carlsbad, CA, USA) and then used to transform *Escherichia coli* XL-1 Blue (Toyobo). The purified plasmids were processed for sequencing by the dideoxynucleotide chain termination method on an ABI PRIZM 3100 Genetic Analyzer (Applied Biosystems, Tokyo, Japan).

### Quantitative RT-PCR

One-step RT-PCR was performed in 20 μl reaction volumes with 1 μg of each RNA template and custom-made primers and probes (Supplementary Table 5) with a TaqMan RNA-to-CT 1-Step Kit (Applied Biosystems, Foster City, CA), in accordance with manufacturer instructions. Quantitative RT-PCR (qRT-PCR) was performed on a Step One plus Real-Time PCR System (Applied Biosystems, Foster City, CA, USA) following the Delta-Delta Ct method. Ribosomal protein 49 (GeneID: 778453) was used as an endogenous reference for the standardization of RNA expression levels, and all data were calibrated against universal reference data. Relative quantification (RQ) values represent the relative expression level against a reference sample. In this study, RQ value was calculated with the RQ value of each SOD expression in the fat body as 1. All samples were assayed in triplicate as technical replications.

### RNA-seq analysis

To examine the expression of each SOD at the individual level, total RNA was isolated from the fat body, midgut, silk gland, Malpighian tubules, and testis of day 3 fifth-instar male larvae of the *B*. *mori* o751 wild-type strain individually using a PureLink® RNA extraction kit (Thermo Fisher Scientific Inc.), in accordance with the manufacturer’s protocol. The quality of RNA was assessed using an Agilent Bio-analyzer 2100 (Agilent Technologies, Santa Clara, CA, USA). Paired-end sequencing cDNA libraries were constructed with 4 μg of total RNA from o751 wild type these samples (n = 3) with a TruSeq RNA Sample Preparation Kit Set A (Illumina Inc., San Diego, CA, USA) according to the manufacturer’s protocol. RNA-seq was performed using a HiSeq. 2500 system (Illumina Inc.). The data quality of the fastq files was verified with the FastQC tool (Babraham Bioinformatics, http://www.bioinformatics.babraham.ac.uk/projects/fastqc/). The 44 M paired-end reads (2 × 150 bp) were mapped to the reference *B*. *mori* genomes available in the Ensembl Genome database^33^ using the Bowtie2 program version 2.3.2 with default parameters^34^. The RNA-Seq by Expectation-Maximization (RSEM) software version 1.3.0 was used for the calculation of expression values in FPKM^35^.

### Induction of oxidative stress by ultra violet radiation

Day 3 fifth instar larvae were treated with ultraviolet (UV) rays using UVL-56 (1350 μW/cm^2^, UVP) for 1, 2, 6 and 12 hours (4.86, 9.72, 29.2 and 58.32 J/cm^2^). We prepared a cardboard box with two stands at either side to position two UVL-56 lamps at the top of the box^11^. Irradiation distance was adjusted to 7.5 cm from the face of the UVL-56 lamps to inside of the plastic box. Silkworm larvae were placed on the inside of the box for UV irradiation. UV irradiation was performed for 1, 2, 6, 12 hours at 25 °C, and then fat body, integument, midgut and Malpighian tubules were dissected. Control groups were kept for 1, 2, 6, and 12 h at 25 °C the same as in the UV irradiation groups but without UV irradiation.

### Determination of LD50 of day 3 fifth instar larvae by rotenone stimulation

To determine the LD50 of day 3 fifth instar larvae by rotenone (Sigma-Aldrich co. ltd. Missoni, USA) stimulation, we injected rotenone intrahemocoelically to larvae weighing 3.5 to 4.0 g using a disposable syringe (Terumo, Tokyo, Japan) with a 30 G needle. Rotenone was dissolved in DMSO (prepared immediately before use and stored in the dark) at 0, 1.25, 2.5, 5.0, 10, 20, 40 mg/g and injected into larvae in a volume of 10 ml/g body weight. In control groups, only DMSO was injected into larvae at a volume of 10 ml/g body weight. The number of dead silkworms after 24 h was counted and the mortality rate (%) = (X/Y) × 100 was calculated, where X = dead larvae in the group and Y = total larvae in the group. The mortality rates were analyzed with Probit analyses^36^ using the Probit Analysis option in the JMP 10.0 software package (SAS Institute Japan Ltd., Tokyo, Japan) to calculate the LD50. We considered the ROT dose that would be most effective in the experimental model with oxidative stress stimulus by JMP simulation.

### Induction of oxidative stress with rotenone injection

Rotenone, prepared at 20 mg/g (LD25), was injected to 10 larvae in a volume of 10 ml/g body weight. In control groups, only DMSO was injected into larvae at a volume of 10 ml/g body weight. And then, fat body and midgut were dissected from day 3 fifth instar larvae, and processed for total RNA isolation and performed quantitative RT-PCR.

### Induction of oxidative stress with *M*. *luteus* injection

Gram-positive bacteria *Micrococcus luteus* (IAM1056) was cultured in Luria-Bertani medium (Wako co ltd. Tokyo Japan). Bacterial cells in the logarithmic growth phase were harvested by centrifugation at 1800 × g for 20 min at 4 °C, washed twice with insect physiological saline (IPS; 150 mM NaCl, 5 mM KCl, and 1 mM CaCl2), and fixed with 4% formaldehyde by gentle shaking for 1 h. The fixed bacterial cells were harvested by centrifugation at 1800 × g for 20 min at 4 °C and washed five times with Clark’s saline (110 mM NaCl, 188 mM KCl, 1 mM CaCl2, 1 mM NaHCO3, 0.07 mM Na2HPO4). Day 3 fifth instar larvae were surface-sterilized by swabbing with 70% ethanol. Then, 5 µl of IPS containing 1 × 10^6^ cells of *M*. *luteus* was injected into the hemocoel. Five microliters of IPS was injected into the hemocoel as a control. Treated larvae were kept at 25 °C, and the fat body from three larvae was collected at 1, 2, 6, and 12 h post-injection. These samples processed to total RNA isolation and performed quantitative RT-PCR.

## Supplementary information

Supplementary information

## Data Availability

The nucleotide sequences for BmSOD4, BmSOD5, BmSOD6 and BmCCS were submitted to DDBJ/ENA/GenBank (Accession Nos. LC229590, LC229591, LC229592 and LC229593 respectively). The RNA-seq reads supporting the conclusions of this article are available in the Sequence Read Archive (SRA) with accession number: DRA005878 and DRA005094.
