# Supplementary material for: Comparative analysis of seven types of superoxide dismutases for their ability to respond to oxidative stress in Bombyx mori
Source: Sci Rep. 2019 Feb 18;9:2170. doi: 10.1038/s41598-018-38384-8 (PMC6379424; doi:10.1038/s41598-018-38384-8)

Comparative analysis of seven types of superoxide dismutases for their ability to respond to oxidative stress in *Bombyx mori*

Yuta Kobayashi[1‡], Yosui Nojima[2‡] Takuma Sakamoto[2], Kikuo Iwabuchi[1], Takeru Nakazato[3], Hidemasa Bono[3], Atsushi Toyoda[4], Asao Fujiyama[4], Michael R. Kanost[5] and Hiroko Tabunoki[1†]

[1]Department of Science of Biological Production, Graduate School of Agriculture, Tokyo University of Agriculture and Technology, 3-5-8 Saiwai-cho, Fuchu, Tokyo 183-8509, Japan.

[2]Department of United Graduate School of Agricultural Science, Tokyo University of Agriculture and Technology, 3-5-8 Saiwai-cho, Fuchu, Tokyo 183-8509, Japan.

[3]Database Center for Life Science (DBCLS), Joint Support-Center for Data Science Research, Research Organization of Information and Systems (ROIS), Yata 1111, Mishima, Shizuoka 411-8540, Japan.

[4]Center for Information Biology, National Institute of Genetics, Yata 1111, Mishima, Shizuoka 411-8540, Japan.

[5]Department of Biochemistry and Molecular Biophysics, Kansas State University, 141 Chalmers Hall, Manhattan, KS 66506-3702, USA.

e-mail address

Yuta Kobayashi: irukano841@gmail.com

Yosui Nojima: yosui.nojima@gmail.com

Takuma Sakamoto: s154883v@st.go.tuat.ac.jp

Kikuo Iwabuchi:kikkuo@cc.tuat.ac.jp

Takeru Nakazato: nakazato@dbcls.rois.ac.jp

Hidemasa Bono: bono@dbcls.rois.ac.jp

Atsushi Toyoda: atoyoda@nig.ac.jp

Asao Fujiyama: afujiyam@nig.ac.jp

Michael R. Kanost: kanost@ksu.edu

Hiroko Tabunoki: h_tabuno@cc.tuat.ac.jp

‡Authors equally contribute in this study

†To whom correspondence should be addressed. Tel: +81-42-367-5613; Fax: +81-42-367-5613;

E-mail: h_tabuno@cc.tuat.ac.jp

Supplementary information

Results

Manduca sexta SODs**-** *Manduca sexta* have the five types of SODs—MsSOD1, MsSOD2, MsSOD3, MsSOD5, MsCCS1, and MsCCS2—so named based on the homology with BmSODs (Supplementary Table 2 and Supplemantary figure 5). The FPKM value showed MsSOD1, MsSOD2, and MsSOD3 gene as expressed mainly in the fat bodies, midguts, and Malpighian tubules in the larval pre-wondering stage, while MsSOD5, MsCCS1. and MsCCS2 gene showed minor expressions in the tissues of these larvae (Supplementary Table 3).

Figure legends

Supplementary figure 1. *Bombyx mori* superoxide dismutases (BmSODs) mRNA expressions in ultraviolet (UV)-irradiated integument. (A) BmSOD1, (B) BmSOD2, (C) BmSOD3, (D) BmSOD4, (E) BmSOD5, (F) BmSOD6, and (G) BmCCS mRNA expressions in integuments pooled from larvae subjected to UV irradiation at 1.0 h (n = 3), 2.0 h (n = 3), 6.0 h (n = 3), and 12.0 h (n = 3) and in non-irradiated controls (n = 3 each) were plotted as relative quantification (RQ) values. Error bars indicate the relative minimum/maximum expression levels against mean RQ values. Bmrp49 was used as the endogenous control. Technical replication was performed in triplicate.

Supplementary figure 2. *Bombyx mori* superoxide dismutases (BmSODs) mRNA expressions in ultraviolet (UV)-irradiated midgut. (A) BmSOD1, (B) BmSOD2, (C) BmSOD3, (D) BmSOD4, (E) BmSOD5, (F) BmSOD6 and (G) BmCCS mRNA expressions in midguts pooled from larvae subjected to UV irradiation at 1.0 h (n = 3), 2.0 h (n = 3), 6.0 h (n = 3), and 12.0 h (n = 3) and in non-irradiated controls (n = 3 each) were plotted as relative quantification (RQ) values. Error bars indicate the relative minimum/maximum expression levels against the mean RQ values. Bmrp49 was used as the endogenous control. Technical replication was performed in triplicate.

Supplementary figure 3. *Bombyx mori* superoxide dismutases (BmSODs) mRNA expression in ultraviolet (UV)-irradiated Malpighian tubules. (A) BmSOD1, (B) BmSOD2, (C) BmSOD3, (D)BmSOD4, (E) BmSOD5, (F) BmSOD6, and (G) BmCCS mRNA expressions in Malpighian tubules pooled from larvae subjected to UV irradiation at 1.0 h (n = 3), 2.0 h (n = 3), 6.0h (n = 3), and 12.0 h (n = 3) and in the non-irradiated controls (n = 3 each) were plotted as relative quantification (RQ) values. Error bars indicate the relative minimum/maximum expression levels against the mean RQ values. Bmrp49 was used as the endogenous control. Technical replication was performed in triplicate.

Supplementary figure 4. *Bombyx mori* superoxide dismutases (BmSODs) mRNA expressions in rotenone injected midgut. (A) BmSOD1, (B) BmSOD2, (C) BmSOD3, (D) BmSOD4, (E) BmSOD5, (F) BmSOD6, and (G) BmCCS mRNA expressions in midgut pooled from larvae subjected to rotenone treatment at 1.0 h (n = 3), 2.0 h (n = 3), 6.0 h (n = 3), and 12.0 h (n = 3) and in controls (n = 3 each) were plotted as relative quantification (RQ) values. Error bars indicate the relative minimum/maximum expression levels against the mean values. Bmrp49 was used as the endogenous control. Technical replication was performed in triplicate.

Supplementary figure 5. Putative domain structure of MsSOD1 to 5 and MsCCS. Upper digits were shown amino acid position. Sod_cu and Sod_Fe mean distinguishing SOD domains. Yellow box showed signal peptide.

Supplementary figure 6. MsSODs mRNA expression in *M.luteus* injected fat body. The data obtained from *M.sexta* database, and plotted the graph. The expression level was shown as FPKM value in each MsSOD.

Supplementary Table 1. The amino acid sequences of SODs employed in the phylogenic tree.

Organism species	Gene IDs
Homo sapiens	ENSP00000270142,ENSP00000404804,ENSP00000371554, ENSP00000307870
Mus musculus	ENSMUSP00000023707,ENSMUSP0000007012, ENSMUSP00000098768, ENSMUSP00000035486
Danio rerio	ENSDARP00000064375,ENSDARP00000062555, ENSDARP00000097881, ENSDARP000000120216
Xenopus laevis	ENSXETP00000015994, ENSXETP00000045789, ENSXETP0000004537, ENSXETP00000058685
Arabidopsis thaliana	NP_565666.1,NP_563910.2.,NP_001031030.1,NP_001031029.1, NP_172360.1,NP_001077494.1,NP_197311.1,NP_001119245.1, NP_187703.1,NP_001030670.1,NP_849440.1,NP_849441.1, NP_194240.1,NP_001031710.1,NP_197722.1,NP_199923.1, NP_191194.1, NP_001190834.1
Oryza sativa	NP_001062514.1,NP_001050118.1,NP_001053613.1, NP_001060564.1,NP_001055195.1,NP_001056612.1,NP_00105 6774.1
Drosophila melanogaster	FBpp0075958, FBpp0305736, FBpp0290166, FBpp0305282, FBpp0088877, FBpp0088878, FBpp0100120
Aedes aegypti	AAEL006271, AAEL011498, AAEL012368, AAEL014091, AAEL000274, AAEL000259, AAEL002559, AAEL014741, AAEL014742, AAEL017480, AAEL004823, AAEL005108
Anopheles gambiae	AGAP005234, AGAP007026, AGAP007497, AGAP001623, AGAP010347, AGAP010517
Apis mellifera	GB10133,GB14210,GB14567,GB14346,GB19399
Plutella xylostella	PXUG_V1_007360, PXUG_V1_007361, PXUG_V1_075572, PXUG_V1_011049, PXUG_V1_007621, PXUG_V1_078561, PXUG_V1_016319, PXUG_V1_031750, PXUG_V1_007562, PXUG_V1_019318, PXUG_V1_047656, PXUG_V1_006489, PXUG_V1_080226
Manduca sexta	Msex2.00386, Msex2.11087, Msex2.10554, Msex2.12155, Msex2.11085, Msex2.03430
Tribolium castaneum	TC007011, TC010027, TC11770, TC011676, TC011675, TC005780

Supplementary Table 2. Assignment of *M.sexta* SODs

Gene name	Manduca gene ID
MsSOD1	Msex2.00386
MsSOD2	Msex2.03430
MsSOD3	Msex2.11087
MsSOD5	Msex2.11085
MsCCS1	Msex2.12155
MsCCS2	Msex2.10554

Supplementary Table 3. FPKM value of of MsSODs in the pre wondering stage.

Gene	Fat body	Midgut	Malpighian tubules
MsSOD1	111	181	743
MsSOD2	846	158	7
MsSOD3	145	160	4
MsSOD5	0	1	2
MsCCS1	1	5	0
MsCCS2	0	1	214

Supplementary Table 4. Primers used for cDNA cloning

Gene	Forward	Reverse
BmSOD4	5’-TTGAATTCACGTGAGATACCTGGC-3’	5’-CAAATAAACTTAATTTAAGAACAG-3’

BmSOD5-A	5’-TGA TTGAGCATCATTTGTCTTGTAAG-3’	5’-TGAACACAATATACATATGTAGATGG-3’
BmSOD6	5’-ATGCCAAAGTTATTTTGGAT-3’	5’-TCACAAGTATCTTCTTATTA-3’
BmCCS	5’-TTGAGAAATAGCGCTACTCAGCTG-3’	5’-ACACGTGCCCCTACACTTTACAGC-3’

Supplementary Table 5. Primers and probes used for quantitative RT-PCR

Gene	Forward primer	Reverse primer	Probe
BmSOD1	5’-ATCATGGTGGTCCCAGTTCTG-3’	5’-CAGAGTCTTCAATTGCCTCAATGT-3’	5’-FAM-TACGCCATGTCGGCGACCTCG-TAMRA-3’
BmSOD2	5’-TCAATGGTGGTGGTCACATCA-3’	5’-AGGCTTGCCACCATTTGG-3’	5’-FAM-CCACTCGATCTTTTGGCACAACCTGTC-TAMRA-3’
BmSOD3	5’-GCGGCGGTTGTGTATCG-3’	5’-GGATGACCGTGGTCCTTATGTT-3’	5’-FAM-CTGGATCTCATTTCAATCC-MGB-3’

BmSOD4	5’-ACGTGTAGCTTGCGGAGTCA-3’	5’-AACGATGTCACGGCTGAATTC-3’	5’-FAM-CGGTATTAGATCACCAGTTG-MGB-3’
BmSOD5	5’-TCCTCGCGACCAGATTCG-3’	5’-TTCCTCCGCCTTGATGTTG-3’	5’-FAM-CACGTTGGTGACCTC-MGB-3’
BmSOD6	5’-CCCAGCGCTCCCCATT-3	5’-TGTCGGAAGCACCAAGTTGA-3	5’-FAM-CCGACCTGGGTTAAC-MGB-3
BmCCS	5’-GGCCGTCGCCATGGT-3’	5’-ACGCCCAGGATCTTATTGCA-3’	5’-FAM-TCGGACCAATCGTG-MGB-3’
Bmrp49	5’-CCAACATTGGTTACGGTTCCA-3’	5’-CATTGTGAACTAGGACCTTACGGAAT-3’	5’-FAM-CAAGAAGACCCGTCATATGCTCCCAAATG-TAMRA-3’

Supplementary figure 1

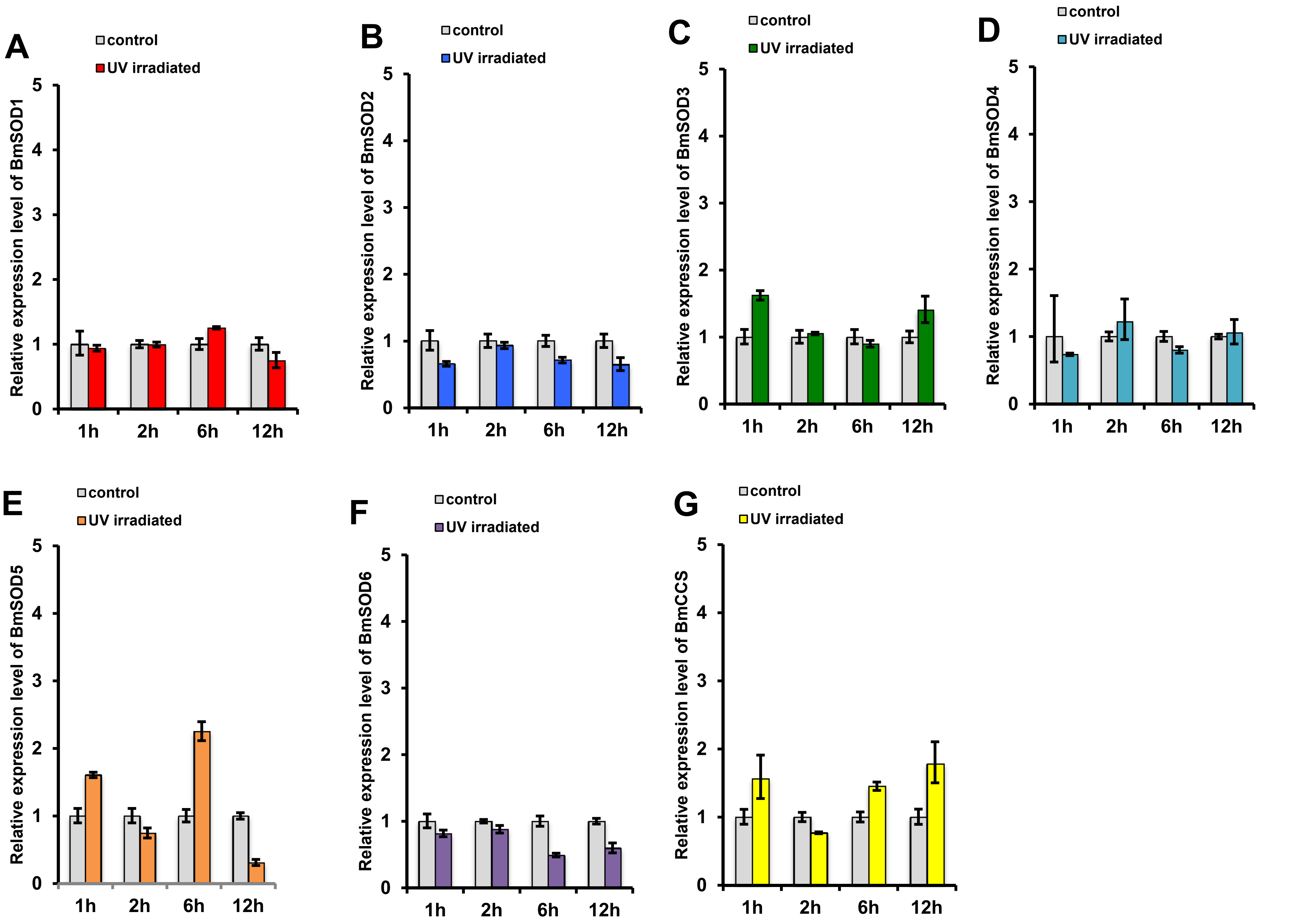

Supplementary figure 2

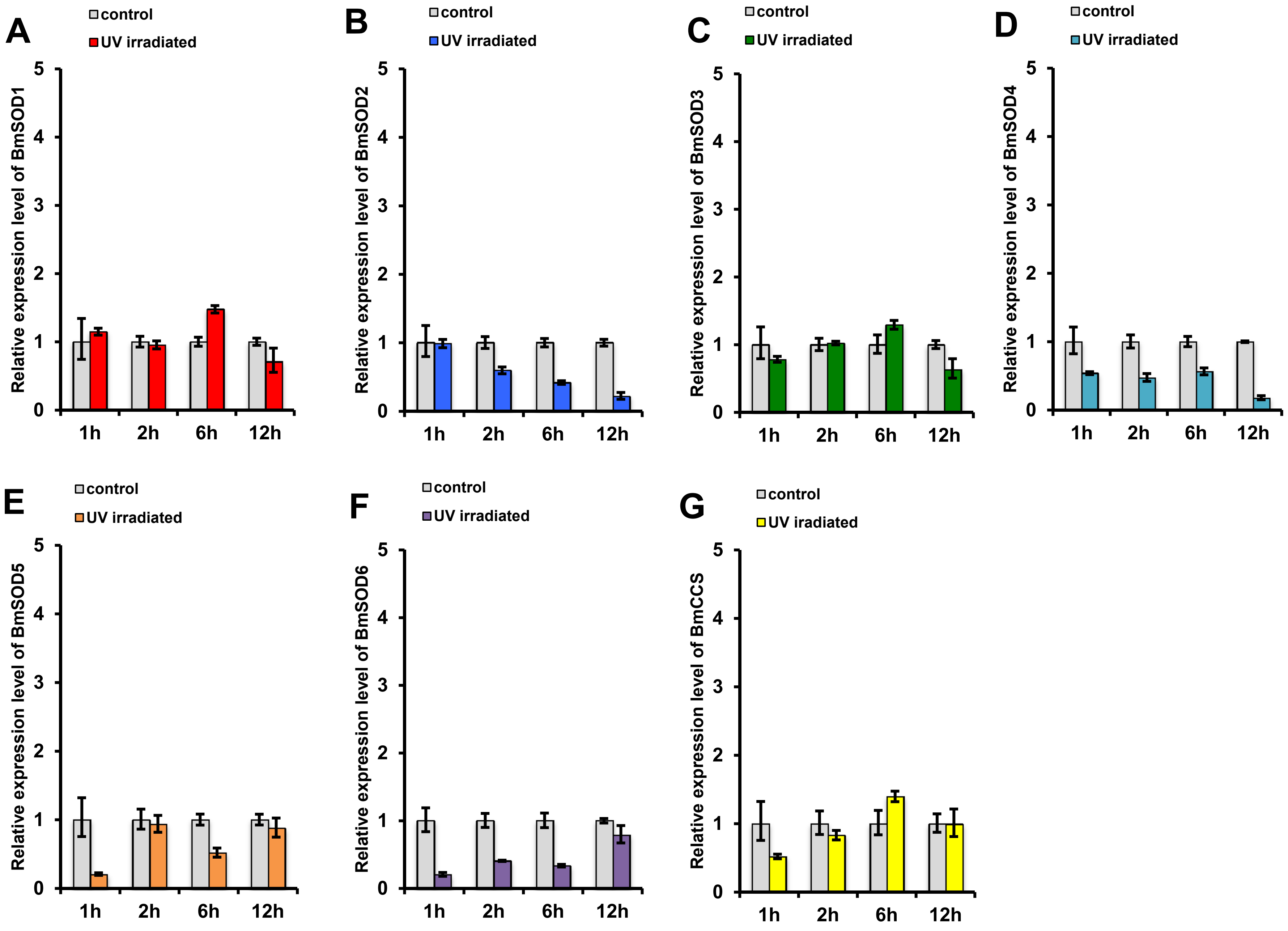

Supplementary figure 3

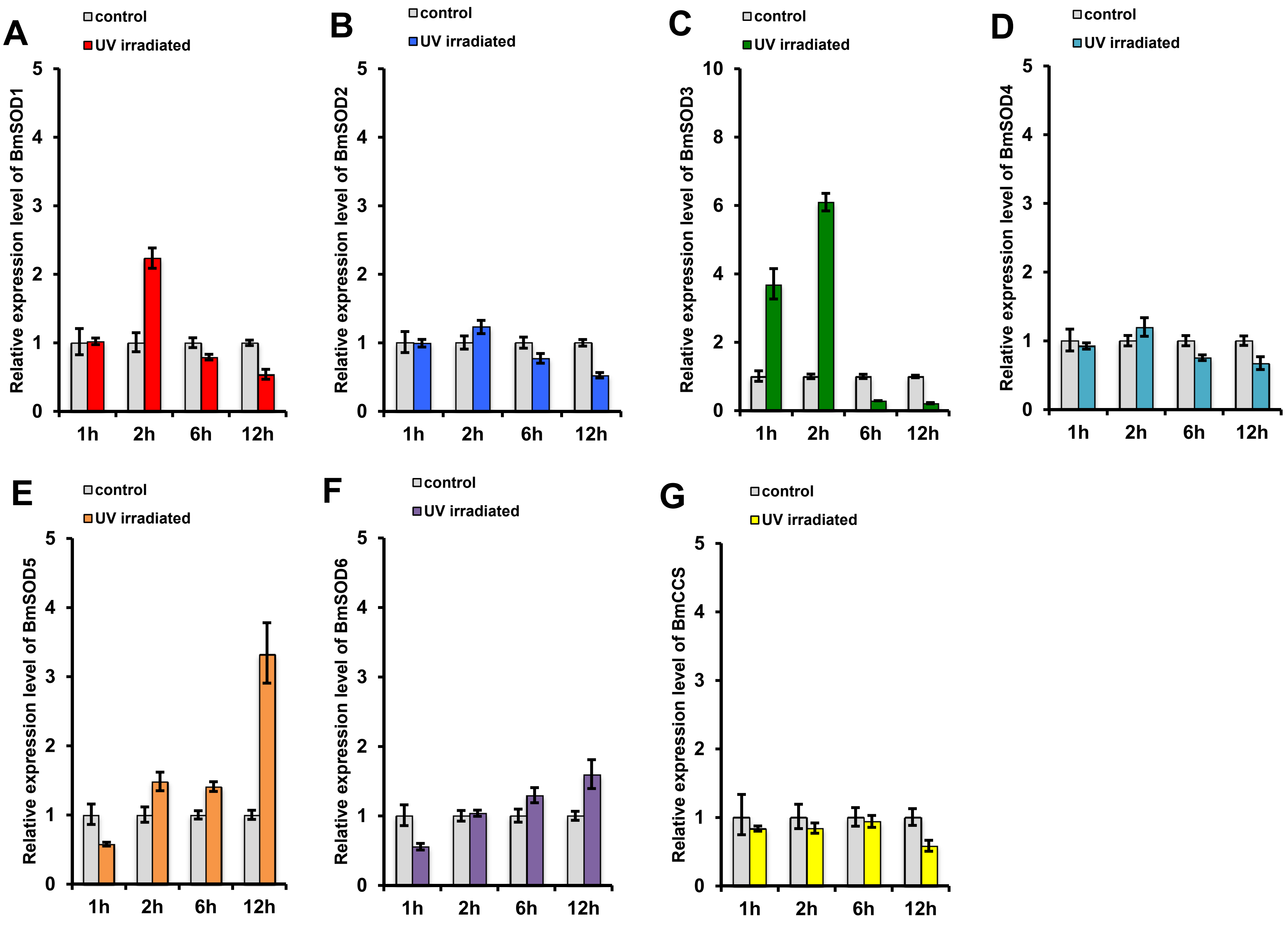

Supplementary figure 4

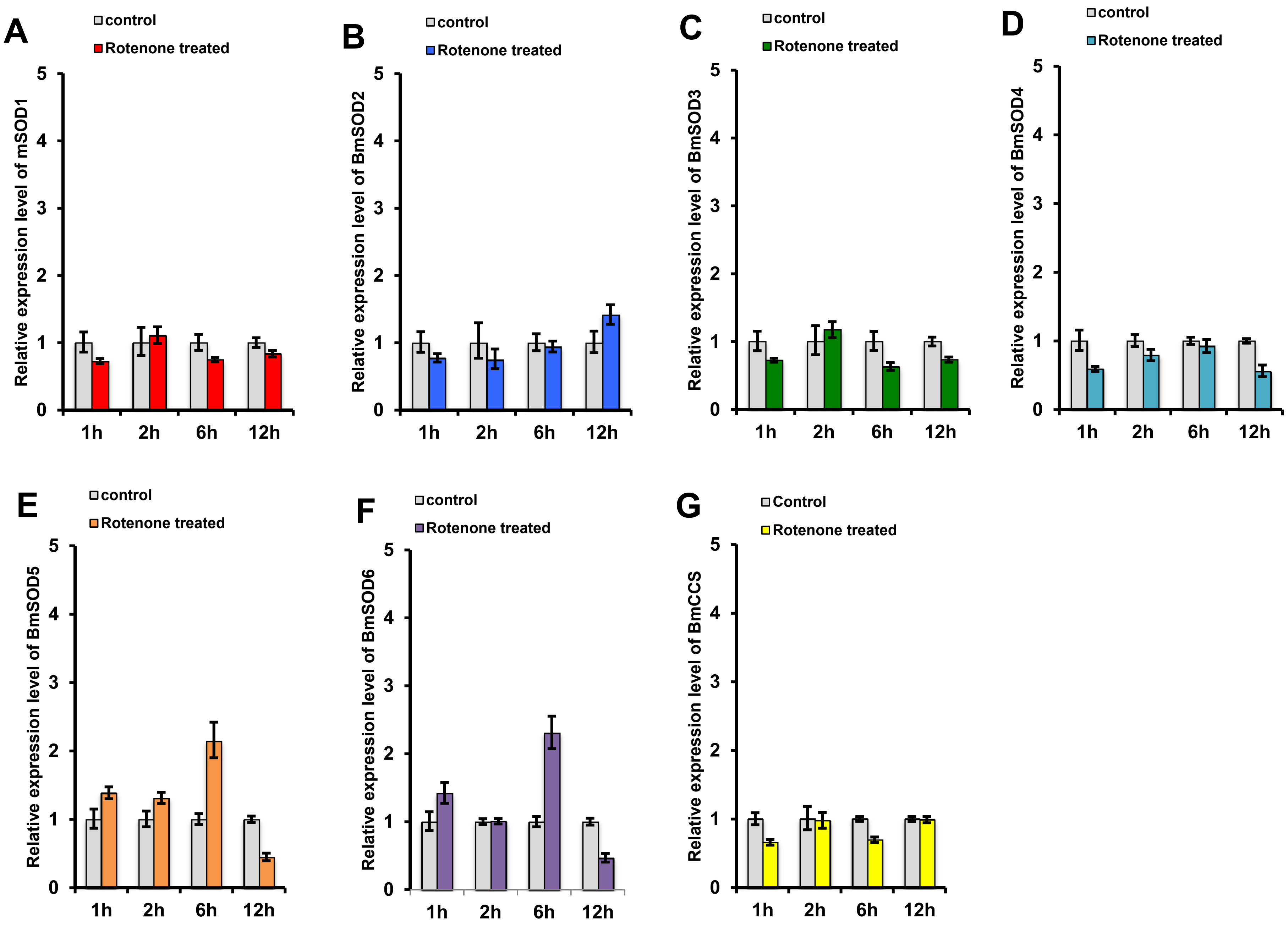

Supplementary figure 5

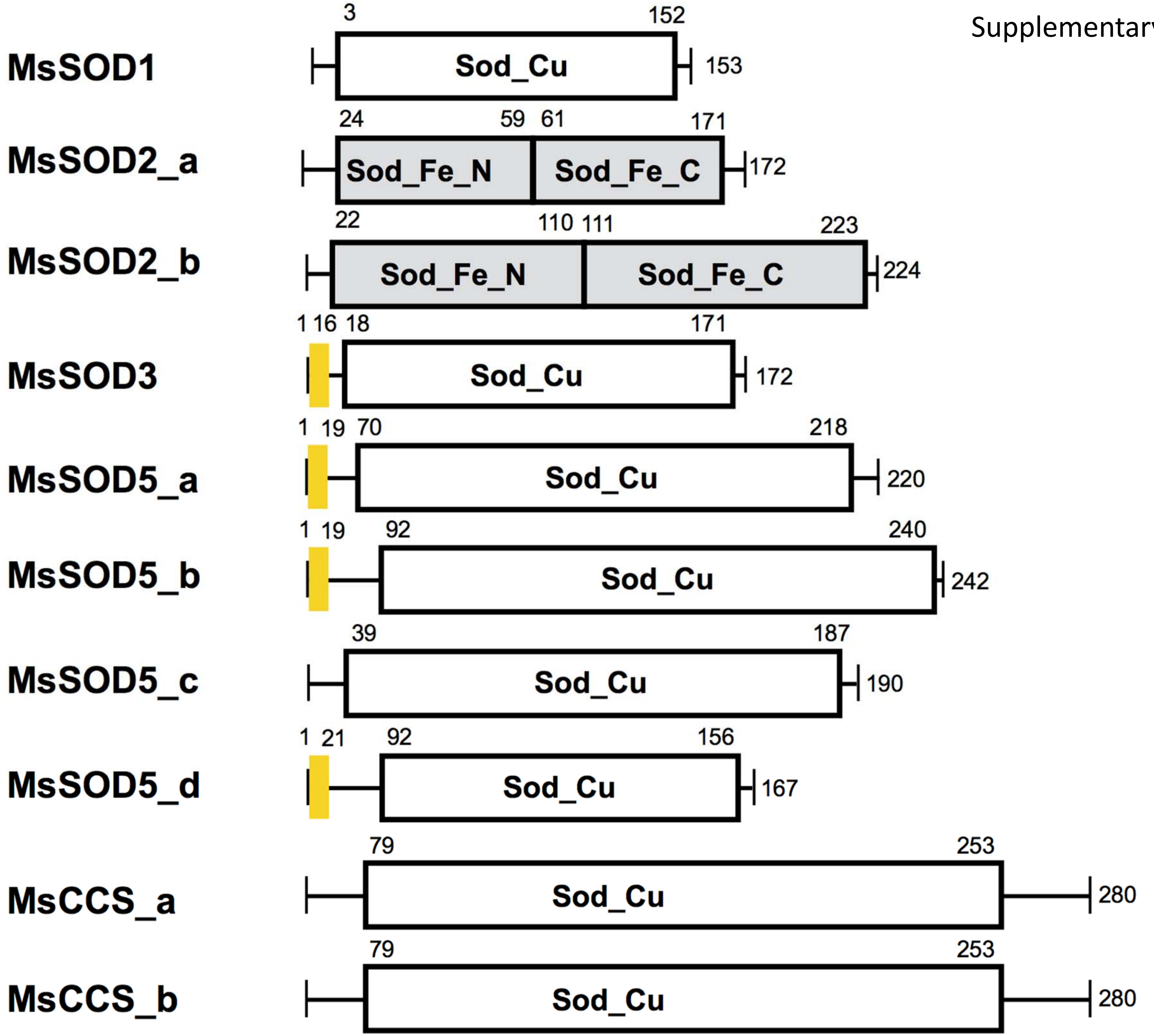

Supplementary figure 6

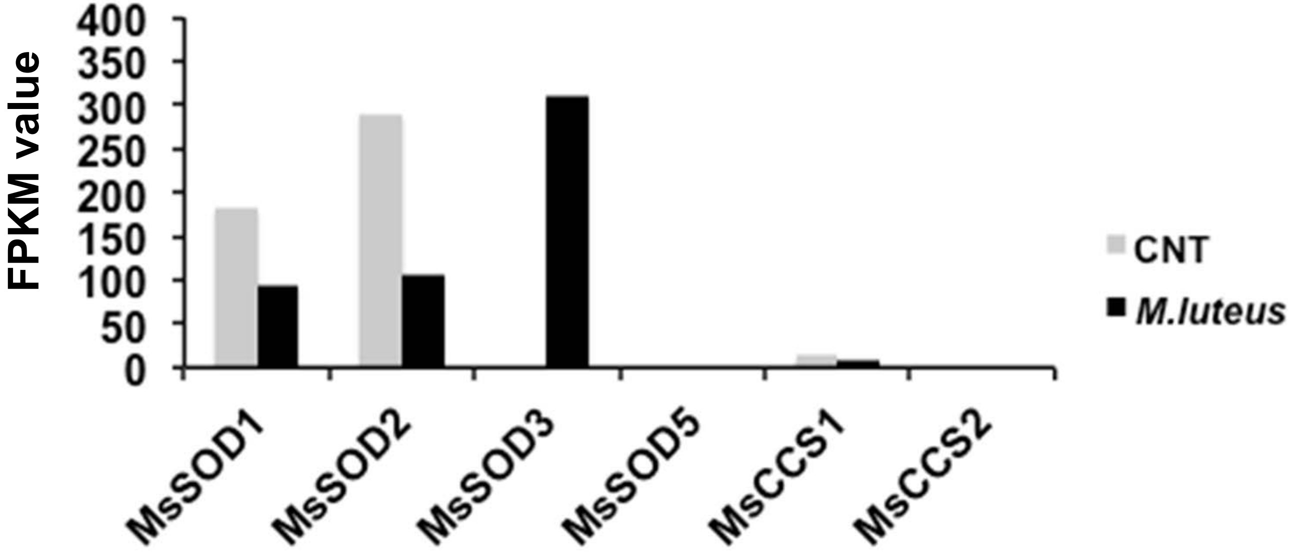

Supplement: Supplementary file 1 — Supplementary information [file 41598_2018_38384_MOESM1_ESM.pdf]
